# Conditions for the Preparation of Self-Compacting Lightweight Concrete with Hollow Microspheres

**DOI:** 10.3390/ma16237288

**Published:** 2023-11-23

**Authors:** Aleksandr Sergeevich Inozemtcev, Sergey Dmitrievich Epikhin

**Affiliations:** Scientific and Educational Center Nanomaterials and Nanotechnologies, National Research Moscow State University of Civil Engineering, 129337 Moscow, Russia; sergep97@mail.ru

**Keywords:** self-compacting concrete, lightweight concrete, structural lightweight concrete, mobility, strength, homogeneity, hollow microspheres

## Abstract

Producing self-compacting concrete with lightweight aggregates is a difficult task. Mixtures with a high content of expanded aggregate tend to separate. It is possible to evaluate the possibility of producing self-compacting lightweight concrete with low average density. This work presents the results of a study of self-compacting lightweight concrete on hollow microspheres. The ability of a lightweight concrete mixture on hollow microspheres with low density (ρ = 1450 ± 25 kg/m^3^) to self-compact has been established. The closeness in the values of the spreading diameter before and after shaking on the table *D*_sp,1_ → *D*_sp,2_ confirms this. The dependences (regression equations) of mobility, coefficients of the Ostwald–Weil equation, and density and strength on the W/C ratio and plasticizer concentration for lightweight concrete with a volume content of hollow microspheres of 46.4% have been established. The limits for homogeneity of lightweight concrete mixtures on hollow microspheres are W/C ≤ 0.6 and C_Pl_ ≤ 1.0%. The dispersion of quartz sand (varying the S_p_/S_f_ ratio) in an amount of 8.7% in the composition of lightweight concrete does not have a significant effect on the self-compaction criterion and physical and mechanical properties. Changes in the physical and mechanical properties of lightweight concrete on hollow microspheres in the selected range of varying the W/C ratio and plasticizer concentration are in the following ranges: ρ = 1403–1485 kg/m^3^, *R*_fl_ = 3.34–5.90 MPa, *R*_com_ = 29.6–45.7 MPa. The presence of delamination at W/C ≥ 0.6 does not allow one to correctly establish the influence of variable factors.

## 1. Introduction

Considerable efforts and investments are being directed toward the search for new technologies for modern methods of construction [1]. The studied technologies in the field of construction material science are being modernized [2]. Thus, the study and development of new technological approaches to the production and selection of components to obtain materials with increased requirements for physical, mechanical, and operational properties has relevance [1,2]. The study of self-compacting concrete (SCC) has been of interest since the end of the last century [3,4,5]. This material is interesting because of its ability to fill the space inside the formwork without compaction while maintaining uniformity [1]. This simplifies the technology for manufacturing reinforced concrete products [4].

Currently, the process of obtaining and using SCC is very extensive [1,2,3,4,5,6,7,8]. The composition of heavy self-compacting concrete includes aggregates with optimized sizes, effective polycarboxylate plasticizers, and mineral additives of various natures [6,7]. There are examples in the literature wherein secondary raw materials were used as a filler for SCC [8]. It has been shown that replacing natural fillers makes it possible to obtain mixtures with varying rheological properties. Rheological properties depend on many factors, including particle size, surface roughness, and filler porosity [9]. The authors of [9] found that the content of recycled aggregates allows one to control the yield strength and plastic viscosity in a self-compacting concrete mixture. This mechanism is explained by an increase in friction between particles, a decrease in the effective liquid content (the surface is rough), and the presence of pores.

The most common fillers for reducing the density of concrete are pumice, rice husk, diatomite, sawdust, volcanic cinders, scoria, sintered fly ash, artificial cinders, expanded perlite, bloated clay, coke breeze, and exfoliated vermiculite [10,11]. However, producing self-compacting concrete using lightweight aggregates is associated with natural difficulties. The decrease in the average density complicates the possibility of obtaining such concrete [11,12,13]. The main problem is the increased tendency to stratify this system. The reviews by the authors of [11,14,15,16,17] made it possible to analyze the work on studying the influence of lightweight aggregates on the technological and physical–mechanical properties of lightweight self-compacting concrete. The concentration of water; the binder; and the introduction of a pozzolan additive, fibers, or nanomaterials has the same effects on the LWSCC and on the SCC [1,2,10,11,14]. Nanosilica, nanoscale titanium oxide, and fiber are used to improve the strength characteristics of lightweight concrete [15]. Filling porous aggregate with water, polymers, or liquid glass is a traditional way to improve the technological properties of a concrete mixture. The average density of LWSCC over 1200 kg/m^3^ usually meets the requirements for use as a structural material [16]. The authors of [18] described the experience of preparing lightweight self-compacting concrete compositions on expanded clay aggregate without segregation or flotation. This paper proposes an effective combination of expanded clay of various fractional compositions (breakstone and sand with an average particle diameter of 9.5 and 4.5 mm, respectively). The content of the mortar part of 60% of the volume of the entire mixture ensures the prevention of the flotation of lightweight filler. It was noted that the amount of lightweight sand and binder in the composition of the mixtures has a negative effect on the self-compacting coefficient. In [19,20,21], the problem of delamination of self-compacting concrete mixtures on lightweight aggregates was solved by introducing fiber. At the same time, dispersed reinforcement also compensates for the decrease in strength and deterioration of crack resistance in concrete with reduced density. The composition of plasticized cement–mineral matrices (with ash and silica) with metal fibers with varying geometries demonstrates satisfactory spreading under filling by quartz sand and expanded clay crushed stone but worse than heavy concrete compositions [20].

Rheological and technological characteristics and production features are the main indicators of quality for concrete mixes. An analysis of these key factors is presented in [2,22,23,24,25,26]. It was found that the types of cement, aggregates, additives, and fibers have a significant impact on rheology. The main quality parameters for SCC have also been set. Traditionally, shear yield strength (t_0_) and plastic viscosity (µ) have been used to describe the rheology of self-compacting concretes. The thixotropy of the mixture is another important property that determines the ability of the system to maintain its uniformity [23]. The use of fly ash with a low calcium content (less than 10%) and finely granulated blast furnace slag contributes to reducing these indicators [22,26]. This is because of a spherical particle ball bearing and the lower chemical activity of the smooth surface of the grains compared to Portland cement, respectively. The reverse effect is observed when using fly ash with a high content of calcium oxide. This is associated with irregular particle sizes and cellular structures. The introduction of fibers of various types, sizes, shapes, and quantities contributes to an increase in yield strength and plastic viscosity. The use of plasticizing additives naturally reduces the value of the rheological parameters [27].

A natural limitation to reducing the density of concrete is the density of the aggregate [14,20,28]. Hollow microspheres are a component of concrete that allow you to solve this problem because they have a lower density than expanded clay, for example. The effectiveness of using hollow glass or ceramic micro-sized particles to produce concrete is shown in [28,29,30,31,32,33]. There is a precedent [28] for producing high-strength lightweight concrete compositions based on hollow microspheres with a specific strength of up to 50 MPa. The positive effect of hollow microspheres on the thermal conductivity of lightweight concrete has also been shown. Concrete made with alternative expanded aggregates has lower specific strength [11]. However, the mobility of concrete mixtures on hollow microspheres is limited due to the tendency for delamination [29,33]. In this case, the intensity of delamination is proportional to the decrease in density. As a result, limiting the density of composites solves the problem of producing self-compacting concrete on hollow microspheres, and compositions with a high content of light aggregate do not demonstrate uniformity.

A study on the rheological characteristics of a concrete mixture that had partially replaced cement with hollow glass microspheres was carried out in [33]. It was found that the concrete mixture, which contained hollow glass microspheres (5% of the binder weight), had a higher static and dynamic yield strength and lower viscosity than the control mixture (without microspheres). The beginning of the flow of such a concrete mixture and its maintenance occurs at higher shear stresses. However, replacing cement with hollow glass microspheres leads to a 15% decrease in the strength characteristics of concrete. The exception is the tensile strength. However, the author does not indicate the average density of concrete [33]. Hollow microspheres in an amount of 5% are only a modifier of rheological properties. This is what the author shows.

It has been shown [34,35] that compositions based on hollow microspheres with low average density (less than 1800 kg/m^3^) and high strength are effective structural materials. The effectiveness of the physical and mechanical properties of such concrete for the construction of multi-story buildings or long-span structures is substantiated in [28]. Violation of the homogeneity of dispersed systems with a light dispersed phase is a limitation to the production of self-compacting lightweight concrete for monolithic construction. Therefore, the development of compositions for self-compacting structural lightweight concrete is a challenging task.

A natural limitation for obtaining self-compacting lightweight concrete mixtures is the presence of a lightweight aggregate, which tends to ascend when critical liquefaction is reached at a certain degree of filling of the dispersion medium [11]. At the same time, the concrete mixture is stratified, and its technical characteristics are reduced. The use of hollow microspheres for self-compacting concrete is associated with two problems: an increase in the W/C ratio to increase mobility due to the large specific surface area of the lightweight aggregate and the danger of delamination (floating of microspheres) due to the large W/C ratio [28]. In this regard, establishing the possibility of obtaining self-compacting lightweight concretes on lightweight aggregates with low average density is the main task [10,11,14]. Studying the mobility of lightweight concrete mixtures on hollow microspheres is one of the ways to solve this problem.

## 2. Materials and Methods

The purpose of this study was to evaluate the possibility of producing self-compacting lightweight concrete with a high content of lightweight aggregate. Also, this study is necessary to establish prescription limits for homogeneous compositions of lightweight concrete with an average density of about 1400 kg/m^3^ [28,33,34].

Lightweight concretes with hollow microspheres were used as the object of this study [28,36]. The composition of the experimental concrete mixture includes the following components: Portland cement (PC), aluminosilicate microspheres (MS), complex silica additive FremSilica-2 (SA), fractional sand (S_f_), quartz powder (S_p_), plasticizer (Pl), and water (W). The ratio of dry components (PC:MS:S_f_:S_p_) was constant and corresponded to the average density of concrete according to the project (1400 kg/m^3^). The volumetric content of lightweight aggregate was 0.464.

Ordinary Portland cement—CEM I 42.5 N manufactured by “Maltsevsky Portland Cement” (Fokino, Bryansk region, Russia) [37]—was used as a binder. The chemical and mineralogical compositions are presented in Table 1. The main properties of this cement are presented in Table 2.

The complex silica additive “FremSilica 2” (LLC “Additives to concrete and pigments “FRAME”, Smolensk, Russia) consists of small particles of amorphous but spherically shaped silica (SiO_2_ > 95%). The average specific surface area is 20 m^2^/g, in accordance with [38]. The average size of one particle is about 0.1 μm.

Aluminosilicate microspheres “ForeSphere” (LLC “ForeSphere”, Ekaterinburg, Russia) were used as a lightweight aggregate to reduce the average density of the concrete. The particle diameter of these microspheres is 10–500 μm. Their main properties are presented in Table 3.

The plasticizer Melflux 2651F (BASF Construction Additives, Trostberg, Germany) was used to control the technological properties of the concrete mixtures. Melflux 2651F is a second-generation polycarboxylate ester that does not affect the setting of cement.

Quartz powder is fine sand that is obtained from ground fractional quartz sand (Rusean, Ramensky district, Ostrotsy village, Russia) in a ball mill. The specific surface area of the quartz powder we used was 720 m^2^/kg.

The properties of the object were studied using the method of mathematical experimental planning. For this purpose, the following standard two-factor model was used:

*Y* = *f*(*X*_1_, *X*_2_) = B_0_ + B_1_·*X*_1_ + B_2_·*X*_2_ + B_12_·*X*_1×2_ + B_11_·*X*_1_^2^ + B_22_·*X*_2_^2^.

The search for equation coefficients made it possible to establish the dependence of quality parameters *Y* on variable factors (*X*_1_ and *X*_2_), significantly reducing the number of experiments.

Interdependent classical variable factors such as *X*_1_—the amount of water (W/C ratio)—and *X*_2_—the concentration of the plasticizer (*C*_Pl_, % of PC)—were used to evaluate the technological and rheological properties of the concrete mixture with hollow microspheres. The main levels were 0.5 (*X*_1_) and 1.4 (*X*_2_), and the variation intervals were 0.1 and 0.4, respectively (Table 4).

Compositions of heavy concrete were used as prototypes for a comparative assessment of the properties of the lightweight concretes: Prototype 1—W/C = 0.5, *C*_Pl_ = 1.4%; Prototype 2—W/C = 0.45, *C*_Pl_ = 1.2%. The ratio of components in these concrete mixtures was established in accordance with Table 5.

The studied composition of lightweight concrete includes a mineral phase of varying dispersity. This is fractionated and fine (powder) sand. Varying the ratio of fine and fractionated sand particles (Table 6) makes it possible to study role of specific surface area. The content of all components of the lightweight concrete mixture was constant (Table 5): W/C = 0.5 and *C*_Pl_ = 1.4%. The total content of the quartz components in the lightweight concrete mixture was S_p_ + S_f_ = const = 8.7%.

**Figure 1:**
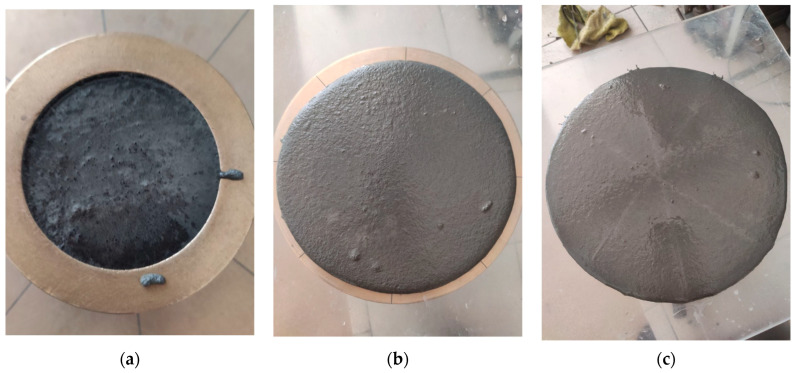
Methodology for determining the mobility of the concrete mixture with hollow microspheres: (**a**) filling the cone; (**b**) spreading diameter after removing the cone (before shaking), (**c**) spreading diameter after shaking.

The mobility of the concrete mixture was determined in accordance with [39] using a shaking table. The spreading diameter was measured based on the truncated cone *D* × *d* × *h*—101.6 × 69.9 × 50.8 mm [40] before and after shaking (Figure 1).

**Figure 2:**
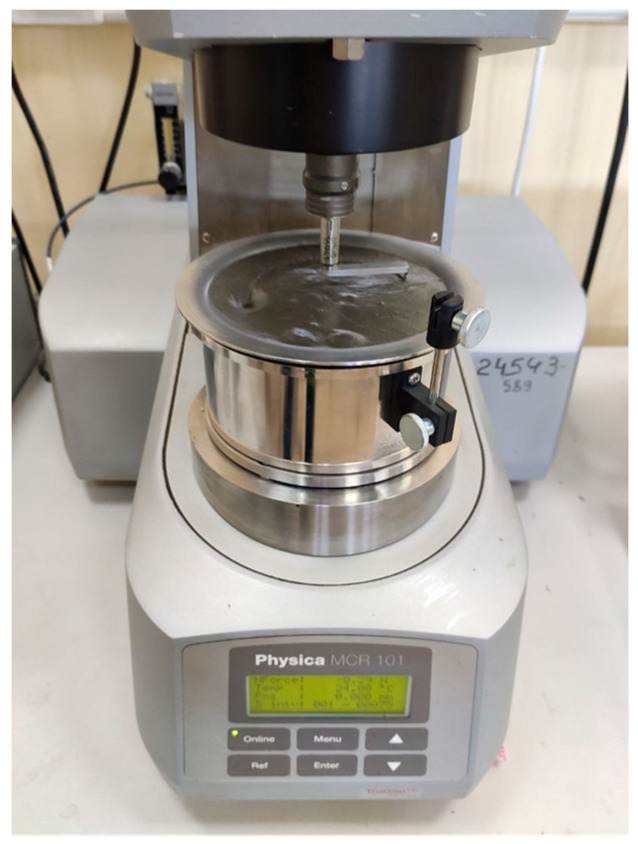
Determination of the shear stress of the concrete mix with hollow microspheres by the rotary viscometer MCR 101.

The rheological properties were studied based on the shear stress of the concrete mixture. The rotary viscometer MCR 101 (Anton Paar GmbH, Graz, Austria) was used for testing (Figure 2). The method used to determine the rheological parameters consisted of measuring the moment of resistance to the movement of a measuring system immersed in a concrete mixture. The measuring system, which is a metal ball with a diameter of 8 mm, moves along the orbit with a shear rate ranging from 0 to 1 s^−1^ for 60 s.

The tests were carried out according to the following algorithm: (1) homogenization of dry components of the mixture; (2) addition of water with dissolved plasticizer; (3) mixing of the concrete mixture according to [41]; (4) filling of the test bowl of the viscometer, positioning of the measuring system in the zero position, stabilization of the system (amount of time = 9 min); (5) measurement. The total test time of the concrete mixture after its preparation was 22 ± 2 min. The number of repetitions of measurements was equal to two. The intermediate stage of homogenization (mixing) was carried out between the first and second measurements. After the test, the concrete mixture was re-homogenized and placed in molds for the manufacture of standard sample prisms 40 × 40 × 160 mm.

**Figure 3:**
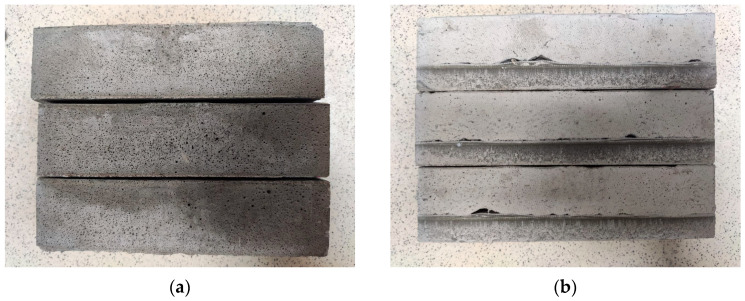
Appearance of lightweight concrete samples with homogeneous (**a**) and stratified (**b**) structures.

Standard samples were used to determine the average density and flexural and compressive strength after 28 days of hardening. A visual analysis of the concrete samples was carried out before determining their mechanical properties. The purpose of this procedure was to establish the presence or absence of stratification of the mixture and the formation of voids in the concrete sample. Examples of samples with signs of stratification, as well as the presence of voids, and samples without these signs are shown in Figure 3.

**Figure 4:**
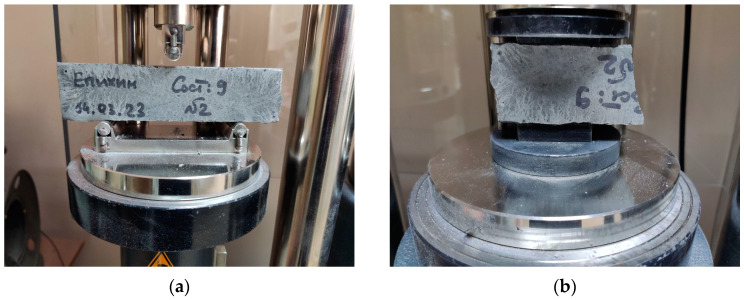
Scheme of testing samples to determine flexural strength (**a**) and compressive strength (**b**).

The Advantest 9 servo-hydraulic system (Controls Group, Milan, Italy) was used to study the physical and mechanical properties (Figure 4) in accordance with [39,42] using three series of samples. Series 1 consisted of samples molded immediately after mixing. Series 2 consisted of samples molded after tests to determine the mobility of the mixture. Series 3 consisted of samples molded after determining the rheological properties.

## 3. Results and Discussion

The technological and rheological properties and homogeneity of the concrete mixtures, as well as the physical and mechanical properties of the hardened composite, were studied for lightweight concrete compositions with hollow microspheres.

### 3.1. Technological Properties

Standard methods for determining the mobility of concrete mixtures allow you to establish a quantitative indicator, such as the spreading diameter from a truncated cone. This indicator is a generally accepted fluidity criterion for mixtures based on mineral binders. For the studied concrete, this characteristic determines the ability to spread under shaking.

Thus, the spreading diameter obtained without shaking should be considered a natural criterion for self-compacting mixtures. Consequently, if the spreading diameter without shaking (*D*_sp,1_) tends to the spreading diameter after shaking (*D*_sp,2_), then, naturally, mobility is taken as the ability of the concrete mixture to self-compact:

*D*_sp,2_/*D*_sp,1_ → 1. (1)

In this case, the intensity of self-compaction will depend on absolute values. A justified prototype for self-compacting lightweight concrete is heavy concrete with an identical composition and target values regarding technological properties but without lightweight aggregate. The determination of the dependence of this criterion on variable factors makes it possible to conclude that it is possible to obtain self-compacting concrete mixtures on hollow microspheres with an average density of 1400 kg/m^3^. It is also possible to determine the boundary ranges for water (W/C ratio) and the concentration of the plasticizer (*C*_Pl_, % of the cement weight) at which this possibility is realized. The experiment was mathematically planned (via a complete two-factor model according to Table 4) to implement this task. The model parameters were described earlier in this paper. The following regression equations were obtained:

*Y*_1_ = 303.7 + 71.1·*X*_1_ + 20.3·*X*_2_ − 15.6·*X*_1_*X*_2_ − 40.9·*X*_1_^2^ − 10.9·*X*_2_^2^, (2)

*Y*_2_ = 308.7 + 63.9·*X*_1_ + 17.7·*X*_2_ − 12.5·*X*_1_*X*_2_ − 31.1·*X*_1_^2^ − 7.75·*X*_2_^2^, (3)

where *Y*_1_—the spreading diameter of the concrete mixture before shaking (*D*_sp,1_), mm; *Y*_2_—the spreading diameter of the concrete mixture after shaking (*D*_sp,2_), mm.

**Figure 5:**
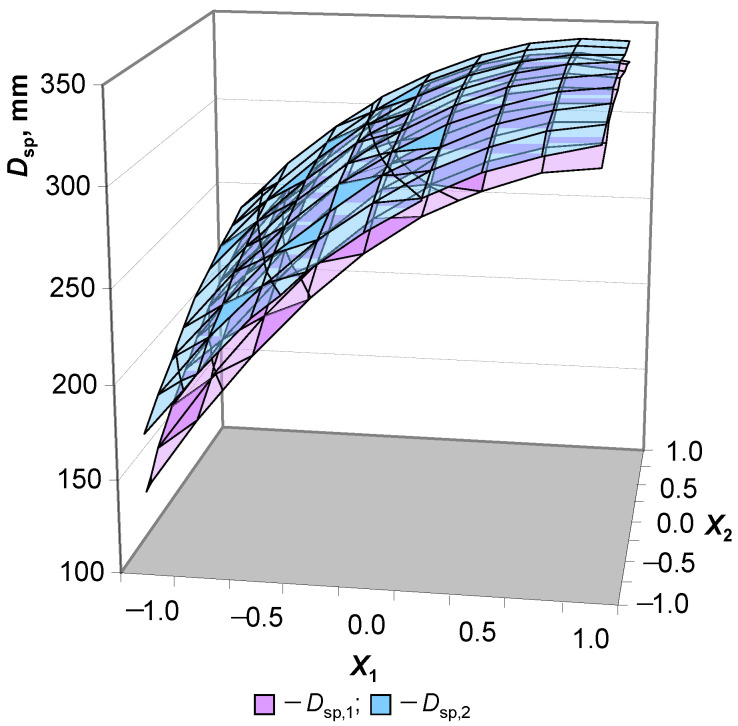
Dependence of the concrete mixture spread diameter on the W/C ratio (X_1_) and plasticizer concentration (X_2_).

The coefficients of the established models for the spreading diameter of the concrete mixture before and after shaking have similar values. The values of the coefficient B_0_ show that the concrete mixture has a close spreading diameter of *D*_sp,1_ ≈ *D*_sp,2_ (differ within 2%) when the variable factors corresponding to the main levels of plan are as follows: W/C = 0.5 and *C*_Pl_ = 1.4%. Each of the coefficients, except B_0_, has a greater value in modulus in the *Y*_1_ model than in the *Y*_2_ model. This indicates a more intense influence of the studied factors on spontaneous spreading than on spreading under the influence of shaking. At the same time, the general nature and direction of the influence of factors for both models are naturally identical. The common extreme value for *D*_sp,1_ and *D*_sp,2_ with a spreading diameter of more than 300 mm is the values *X*_1_ ≥ 0.25 in the entire range *X*_2_ (Figure 5). The ranges of variation for the selected factors make it possible to obtain concrete mixtures on hollow microspheres with high mobility. This is expressed by spreading diameter values of up to 325 mm before shaking and up to 335 mm after shaking.

**Figure 6:**
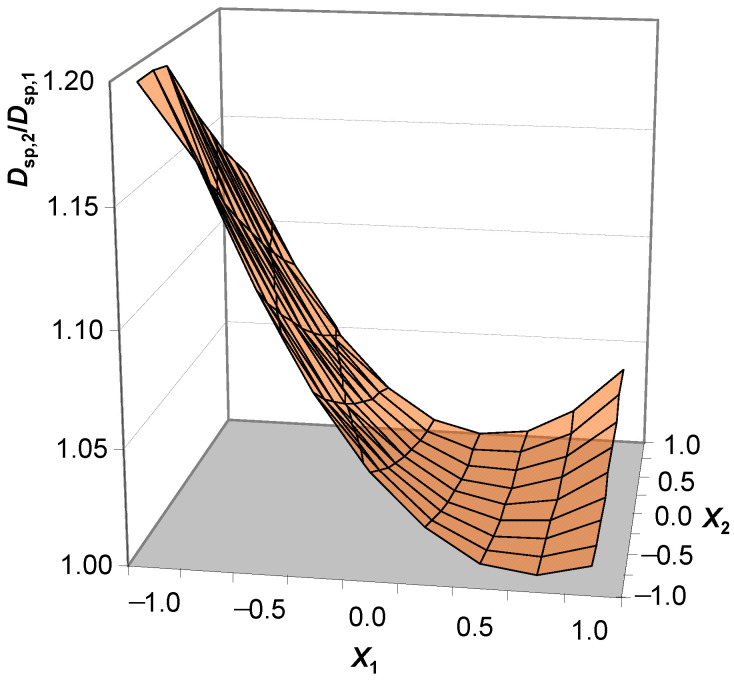
Dependence of *D*_sp,2_/*D*_sp,1_ on the W/C ratio (*X*_1_) and plasticizer concentration (*X*_2_).

Figure 6 shows that, in the studied ranges, external influence leads to insignificant liquefaction after self-spreading (*D*_sp,2_/*D*_sp,1_ < 1.20) in concrete mixtures on hollow microspheres. This indicates the ability of the studied mixtures to spread independently and obtain values close to the maximum values of the spreading diameter. The dependence of *D*_sp,2_/*D*_sp,1_ = *f*(W/C, *C*_Pl_), as shown in Figure 6, shows that compositions with a wider range of factorial variation have the ability to self-compact regardless of the absolute values of the spreading diameters of the concrete mixture, with *D*_sp,2_/*D*_sp,1_ ≤ 1.05 at *X*_1_ ≥ −0.25 in the entire range of *X*_2_. According to the models obtained, this value corresponds to the W/C ratio of 0.475. The parameters that characterize the mobility of the concrete mixture of heavy concrete (prototype) are presented in Table 7.

A comparative analysis of lightweight and heavy concrete mixtures was carried out. The following observations were noted: Firstly, the variation in the W/C ratio and the content of the plasticizer ensures the better mobility of the lightweight concrete mixtures compared to the heavy ones. Secondly, the ability to self-compact in mixtures with hollow microspheres is higher, meaning there is a lower coefficient of *D*_sp,2_/*D*_sp,1_. At the same time, the compositions of the lightweight and heavy concrete mixtures, with the same content of water and plasticizer (Prototype 1: W/C = 0.5, *C*_Pl_ = 1.4%), are different. The *D*_sp,2_ and *D*_sp,1_ of the lightweight concrete mixture are more than those of the heavy concrete mixtures by 8.2 and 2.2%, respectively, and the self-compacting coefficient is lower by 5.6%. An important requirement for self-compacting concrete is uniformity. For example, the separation of water from the mixture was observed in the compositions of prototypes of heavy concrete with a large W/C ratio. The correction of water and plasticizer consumption (Prototype 2, W/C = 0.45, *C*_Pl_ = 1.2%) eliminates this drawback. The established models make it possible to determine that the spreading diameters of a lightweight concrete mix on hollow microspheres are equal to *D*_sp,1_ = 241 mm and *D*_sp,2_ = 255 mm at W/C = 0.45 and *C*_Pl_ = 1.2%. This means that the self-compacting coefficient (*D*_sp,2_/*D*_sp,1_) of a lightweight concrete mix on hollow microspheres is 7.0% less than that of Prototype 2 with the same ratio of control factors. This indicates the greater role of water distribution in self-compacting systems on microspheres compared to quartz sand (without hollow particles). Such patterns depend on the structural parameters of dispersed systems (for example, the thickness of the water layer) and on the properties of the dispersed phase (hollow microspheres and quartz sand).

The composition of the studied lightweight concretes includes a mineral phase of varying dispersity: fractionated (S_f_) and fine (S_p_) quartz sand. It becomes possible to see how the mineral part of an identical composition affects the distribution of water in the dispersed system. The total volume content of the quartz part in a mixture is constant S_g_ + S_f_ = const = 8.7% at a constant consumption of all components of lightweight concrete and W/C = 0.5, *C*_Pl_ = 1.4% (Table 5). The S_g_/S_f_ ratio was varied from 0 to 1 when each sand fraction was varied from 0 to 100%. The technological properties of concrete mixtures with varying ratios of ground and fractionated sand are presented in Table 8.

Table 8 shows that the spreading diameter of the concrete mixture from the cone without shaking for all compositions in the variable range of the S_p_/S_f_ ratio has values of 301–305 mm, and the spreading diameter after shaking is 313–317 mm (a standard deviation of up to ±6 mm). This means that the mobility of the mixture does not demonstrate the role of the dispersion of quartz sand in the studied range. The self-compacting coefficient varies in a statistically insignificant range of 1.03–1.05. The data obtained indicate that the technological criterion for the quality of the mixture does not allow one to make a statistical conclusion about the influence of the dispersion factor of quartz aggregates on the distribution of water in the system.

Thus, the response of the studied concrete mixtures on hollow microspheres to spontaneous spreading with a volume content of 0.464 was shown. The values of spontaneous spreading are close to the values of mobility during forced spreading (after shaking). The ranges of the main recipe factors are established. It is shown that lightweight concrete mixtures can have better technological properties than heavy concrete mixtures with similar water contents. Therefore, the features of the rheological properties of such mixtures are also superior.

### 3.2. Rheological Properties

**Figure 7:**
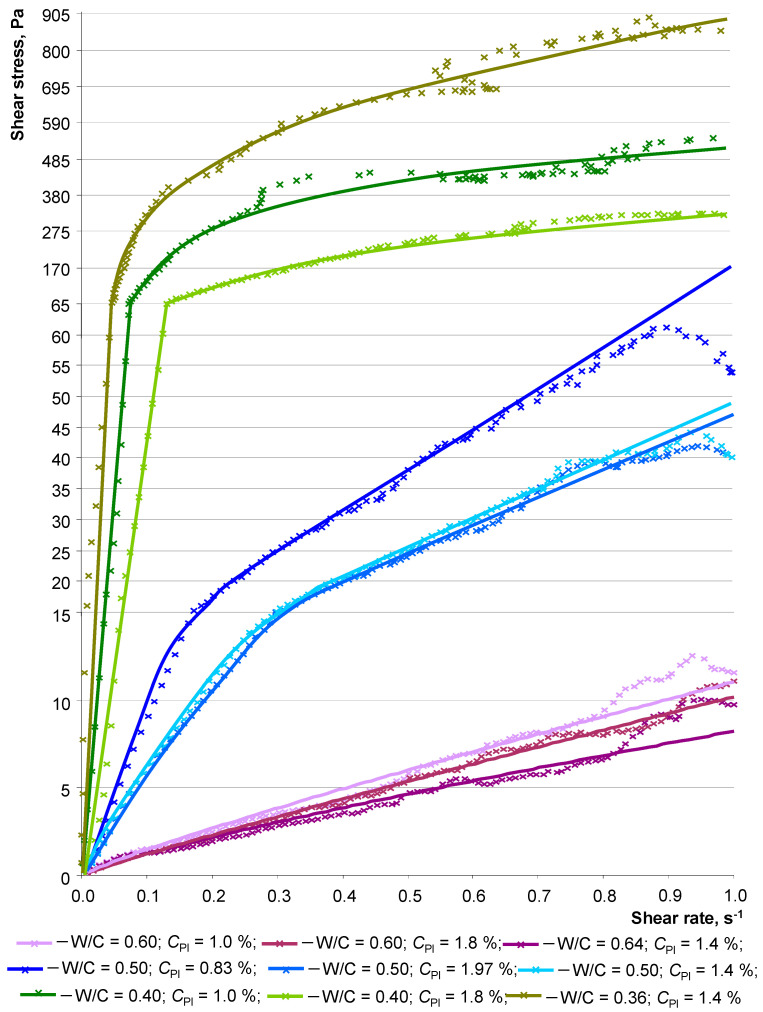
Dependence of shear stress on the shear rate of the concrete mixture when varying the W/C ratio and plasticizer concentration.

For each composition, in accordance with the above experimental plan, rheological curves that describe the dependence of shear stress on shear rate were obtained (Figure 7). The graphs of the rheological curves can be grouped according to the ranges of shear stress in which a change occurs with increasing shear rate. The highest shear stress (τ > 100 Pa) was found in compositions with W/C < 0.4; with W/C = 0.5, the shear stress varies in a range from 40 to 60 Pa, and the third group is formed by compositions with a shear stress that does not exceed 15 Pa.

The graphs show a change in the intensity of the shear stress increment with increasing shear rate. This behavior of the dispersed system is associated with the alignment of structural elements under the action of a shear force and their subsequent compaction due to a decrease in the distance between the grains of the dispersed phase. Following an increase in shear stress after a shear rate of 0.05–0.15 s^−1^, it decreases, and this decrease is associated with overcoming the flow resistance threshold. At this moment, a larger volume of the mixture begins to move. Also, for these compositions, areas of opposite changes in shear stress can be observed under increasing shear rates (Figure 7), specifically at values of about 0.6 s^−1^. This is explained by a violation of continuity due to the formation of voids inside the volume of the mixture. They are formed due to the high density and low ability of the mixture to fill free space.

It is natural that the shear stress increases with a decrease in the W/C ratio, regardless of the concentration of the plasticizer. A similar pattern can be noted for compositions with varying *C*_Pl_. Increasing the plasticizer concentration leads to a decrease in the thickness of the mixture, which is evident from the lower shear stress. In this case, there is a limit for the concentration of the plasticizer, when its amount ceases to affect the rheology of the mixture. Thus, for compositions with a W/C ratio of 0.5, a change in the concentration of the additive higher than *C*_Pl_ > 1.4% is not reflected in the graphs of the dependence of shear stress on shear rate. That is, it is no longer possible to change the distribution of water in the system by increasing the amount of plasticizer. A graphical comparison of concrete mixtures with W/C = 0.5 and *C*_d_ = 1.4% but with different densities showed that the composition without hollow microspheres (Prototype 1) has lower shear stress, and a uniform change in τ indicates the better fluidity and continuity of the system. This may be due to the influence of the light dispersed phase (hollow microspheres), which has a smaller size and density compared to quartz sand. Thus, fine particles of lightweight aggregate require more water, and as we know, the intensity of the flow decreases in proportion to the gravitational effect, depending on the density. However, the composition of heavy concrete with the specified ratio of W/C and *C*_Pl_ is prone to water separation, which indicates its excess. Reducing the value of the variable factors to W/C = 0.45 and *C*_Pl_ = 1.2% made it possible to obtain a prototype of a homogeneous mixture of heavy concrete. Obviously, the shear stress of such a composition is higher and can act as an approximate boundary for obtaining a homogeneous mixture.

The group of concrete mixtures with shear stress values less than 15 Pa included compositions with W/C = 0.6. The shear stress graphs had no discernible differences when varying the plasticizer concentration. A comparative analysis of the obtained rheological curves was performed using the Ostwald–Weil equation

τ = *k*γ*^n^*, (4)

where τ—shear stress; γ—shear rate; *k*—consistency indicator; n—indicator of the flow type (*n* < 1 for pseudoplastic flow, *n* > 1 for dilatant flow). The resulting rheological equations for each composition, according to the experimental plan, were used to establish regression equations *Y* = *f*(*X*_1_; *X*_2_):

*Y*_3_ = 47.0 − 390.9·*X*_1_ − 37.0·*X*_2_ + 64.6·*X*_1_*X*_2_ + 294.3·*X*_1_^2^ + 7.4·*X*_2_^2^, (5)

*Y*_4_ = 0.966 − 0.077·*X*_1_ + 0.029·*X*_2_ − 0.063·*X*_1_*X*_2_, (6)

where *Y*_3_ and *Y*_4_—the coefficient k and n of the Ostwald–Weil equation, respectively.

An analysis of the resulting equations showed that the density coefficient (*k*) has a more significant change from varying the W/C ratio than the additive concentration. At the same time, both factors contribute to a decrease in the value of this criterion. Note the positive sign of the coefficients B_12_, B_11_, and B_22_ in equation *Y*_3_. This indicates a change in the nature of both the mutual influence of *X*_1_ and *X*_2_, as well as a significant increase in their value. That is, both an excess amount of water and plasticizer, and their mutual influence leads to an increase in the density of the concrete mixture. This can be explained by a violation of the homogeneity of dispersed systems when exceeding the threshold number of variable factors leads to an imbalance of water. In this case, water separation is observed. That is, the segregation of the mixture may be associated with a decrease in the thickness of the water layer and an increase in friction between solid particles. Equation *Y*_4_ demonstrates the value of the coefficient *n* < 1 at values of the varied factors corresponding to the main levels of variation (B_0_ = 0.966). That is, such a dispersed system is characterized by a pseudoplastic type of flow. In this case, the concrete mixture begins to flow at minimal shear loads. The shear stress changes with decreasing intensity as the shear rate increases. At the same time, this rheological behavior is enhanced by the w/c ratio, and its combined effect with the plasticizer enhances this rheological behavior of the mixture. This can be seen by the “–” sign in front of the coefficients B_1_ and B_12_ in the regression equation *Y*_4_. At the same time, the coefficients of the equation show the possibility of changing *Y*_4_ to the region *n* > 1. That is, the nature of the flow of the concrete mixture may shift to being dilatant depending on the value of the W/C ratio and the concentration of the plasticizer. In such a system, the cement–mineral paste forms a layer between the particles of the dispersed phase and helps reduce friction. As the shear rate increases, the shear stress changes with increasing intensity. From the equation *Y*_4_ = *f*(*X*_1_; *X*_2_), it is clear that the concentration of the plasticizer (coefficient B_2_) contributes to the tendency towards such behavior of the dispersed system under study.

**Figure 8:**
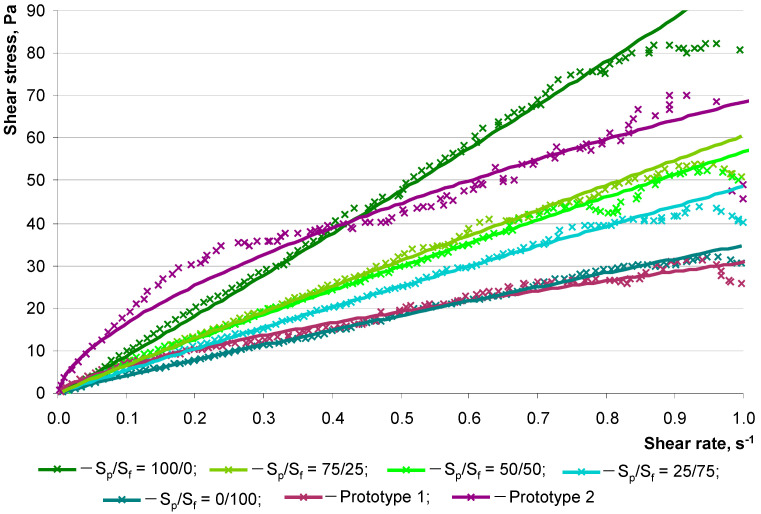
Dependence of shear stress on the shear rate of the concrete mixture when varying the S_p_/S_f_ ratio.

Thus, the obtained regression models show the complex nature of the influence of the studied factors on the rheology of lightweight concrete mixtures. The compositions of concrete mixtures based on hollow microspheres should be optimized according to complex antagonistic indicators. Self-compacting fine-grained heavy concrete can reasonably be considered a target analogue in terms of the nature of the flow of mixtures. Along with the amount of water in a concrete mixture, the most important factor determining its flow pattern is the total surface area of the dry components. The effect of dispersion on the rheological properties of the concrete mixtures under study is demonstrated in Figure 8 and Table 8.

In Figure 8, an increase in the density of the concrete mixture is naturally observed with an increase in the proportion of quartz powder. This can be seen from the proportionally higher values of shear stress. This is also noticeable by the change in the coefficient *k* in the Ostwald–Weil equations for each of the flow curves. Decreasing the proportion of fractionated sand with powder from 100 to 0% helps to increase the density coefficient by almost three-fold (from 35.4 to 102.9) (Table 9). This range is explained by the water consumption for wetting the larger total surface area of the quartz part. In this case, the self-compaction criterion for these compositions changes slightly (*D*_sp,2_/*D*_sp,1_ = 1.03–1.05). The change in the value of the coefficient n indicates a change in the nature of the flow, from pseudoplastic (*n* < 1) to dilatant (*n* > 1), with an increase in the proportion of the thin component (S_f_). This is due to a decrease in the volume of the dispersed phase (fractionated quartz sand) and an increase in the volume of the dispersed medium (cement–mineral matrix).

Let us note the value of the coefficients of the rheological equations for heavy concrete. Compositions Prototype 1 and Prototype 2, with *D*_sp,2_/*D*_sp,1_ = 1.08 and 1.14, respectively, are described by equations with *k* = 31.2 and 77.7 and n = 0.68 and 0.63, respectively. This shows that mixtures of heavy concrete tend to flow at lower values of shear stress than mixtures of lightweight concrete on hollow microspheres. However, the spread diameter of heavy concrete is smaller (Table 7). This is explained by the higher density of dispersed phase particles. The effect of gravitational forces on quartz has a proportionally greater influence. That is, smaller and lighter hollow microspheres require greater force for a similar flow of the dispersed system. Due to the impossibility of realizing this condition, the scientific problem of combining competing rheo-technological properties in concrete mixtures on hollow microspheres is raised. The most important condition for mineral-dispersed systems such as concrete mixtures is homogeneity [43]. Special requirements for maintaining homogeneity should be formulated for self-compacting concrete mixtures based on hollow microspheres. The condition for ensuring the homogeneity of the studied systems with a dispersed phase of different densities (quartz sand and hollow microspheres) can be based on solving the model problem in accordance with Stokes’ law:

(7) $$\upsilon_{s}=\frac{2}{9}\frac{r^{2}\left(\rho_{f}-\rho_{m}\right)}{\mu }$$

where *r* represents the radius of the particle, ρ*_f_* represents the density of the dispersed phase, ρ*_m_* represents the density of the dispersed medium, and *μ* represents the viscosity of the dispersed medium. According to the above formula, the speed of a particle depends on its size and density, as well as the viscosity and density of the medium. Moreover, if ρ*_f_* < ρ*_m_*, then the movement of the particle is directed “up” (towards ascent), while the movement is directed “down” (towards settling) if ρ*_f_* > ρ*_m_*.

**Figure 9:**
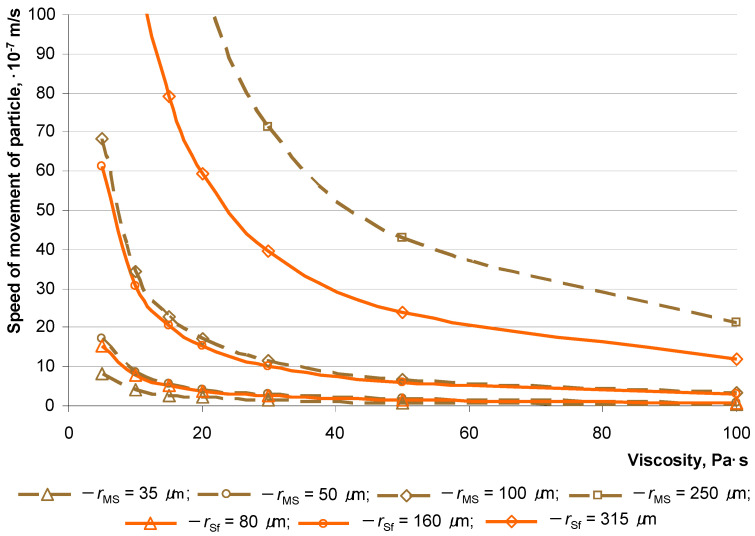
Theoretical dependence of the speed of movement of particles of various sizes and densities on the viscosity of the dispersed medium: solid line for quartz sand (ρ*_f_* = 2650 kg/m^3^); dotted line for hollow microspheres (ρ*_f_* = 540 kg/m^3^).

The concrete studied mixtures are dispersed systems in which the dispersed phase consists of hollow microspheres (average particle diameter no more than 100 microns) and 0.16–0.63 mm fractionated quartz sand, and the dispersed medium is a cement–mineral system that is mixed with an aqueous solution of a plasticizer. The homogeneity of such a system consists of the condition of ensuring a low speed of movement of phases with a known density and geometric dimensions (Figure 9).

The theoretical dependences of the movement speed of particles of different radii and densities on the viscosity of the dispersed medium (ρm = 2100 kg/m^3^) show that lightweight particles (ρ*_f_* = 540 kg/m^3^) with a particle diameter of less than 0.36 mm (*r*_MS_ < 180 μm) have a slower movement speed than the largest particles of fractionated quartz sand (ρ*_f_* = 2650 kg/m^3^, *r*_Sf_ = 15 μm). Thus, with all other things being equal, the movement speeds of particles of different densities can be arranged in the next row of the sequence in accordance with their radius:

*r*_MS_ = 35 μm < *r*_MS_ = 50 μm, *r*_Sf_ = 80 μm < *r*_MS_ = 100 μm, *r*_Sf_ = 160 μm < *r*_Sf_ = 315 μm < *r*_MS_ = 250 μm

That is, hollow microspheres with an average particle diameter of 70–100 µm in a cement–mineral matrix tend to ascend less intensely than quartz sand, and quartz sand with an average particle diameter of 630 µm tends to settle. Thus, the movement speed of particles (the intensity of delamination of the system) can be controlled through the viscosity of the cement–mineral paste through the interdependent factors of the W/C ratio and the concentration of plasticizer *C*_Pl_.

### 3.3. Homogeneity/Segregation

A key requirement for self-compacting concrete mixtures is their ability to maintain homogeneity. Many factors contribute to maintaining the homogeneity of the structure, including the quality and quantity of mixture components, as well as manufacturing conditions and external influences. In the concrete mixtures under study, an additional factor is the different densities of the components of the dispersed phase (hollow microspheres and quartz sand). Therefore, the tendency for lightweight concrete compositions with high self-compacting abilities to delaminate limits their use. According to the experimental plan, samples of each composition were subjected to a visual analysis that involved recording the presence of heterogeneous layers (Table 10).

The data presented in Table 10 show that, at W/C < 0.6 and *C*_Pl_ < 1.0%, there is no delamination on all compositions of Series 1 and Series 2. That is, the compositions molded immediately after preparation (without compaction), and the compositions molded after mobility tests are homogeneous. However, increasing the content of the plasticizer (composition 4) and the amount of water with the plasticizer (composition 6) is sufficient for the separation of concrete mixtures for these series. For Series 3, only compositions 1, 3, and 5 (W/C ≤ 0.4) did not show visual signs of delamination. That is, for compositions 2, 7, 8, and 9 (W/C ≥ 0.5), a change in the structure of the concrete mixture occurs after external influences due to testing. This can be explained by the thixotropic dilution of these concrete mixtures—associated with a decrease in the viscosity of the cement–mineral matrix (dispersed medium) under the action of vibration forces. At the same time, the external effects of the test conducted to determine of the spread diameter do not have such a dilution. The assumption of the presence of a uniformity threshold is derived from this. The structure of the concrete mixture loses its homogeneity after exposure to extreme energy. The results of our visual analysis of concrete samples with varying sand dispersion values are shown in Table 11.

When the S_p_/S_f_ ratio was varied, delamination was observed only in samples of Series 3. This means that, regardless of the dispersion of quartz sand (volume content of 8.7%), samples of the composition of the concrete mixture of the series with W/C = 0.5 and *C*_Pl_ = 1.4% have a similar tendency to delaminate under the influence of external influences caused by testing.

Thus, our visual analysis of the stratification of the samples of the concrete under study showed that compositions with W/C = 0.4 have high structural uniformity, including in the presence of external influences. If W/C = 0.6 and *C*_Pl_ = 1.4%, the concrete mixtures are characterized by a high tendency to segregate. Compositions with W/C = 0.5 with varying *C*_Pl_ values and ratios of fractional sand and quartz powder tend to stratify after a certain level of external influence. For such compositions, it is advisable to use special organic or mineral viscosity modifiers or stabilizers.

### 3.4. Physical and Mechanical Properties

The final product when using self-compacting concrete mixtures is a solid product with specified performance properties. Ensuring the homogeneity of the mixture is an important consideration for obtaining a homogeneous structure and properties in the volume of the solidified composite. In this regard, lightweight concretes with hollow microspheres are complex systems with a defined set of rheo-technological and physico-mechanical properties–antagonists: high mobility and homogeneity, low average density, and high strength.

Regression equations describing the relationship between the W/C ratio and plasticizer concentration with the average density and strength of lightweight concrete on hollow microspheres have been obtained:

*Y*_5_ = 1407 − 26.9·*X*_1_, (8)

*Y*_6_ = 3.42 − 0.8·*X*_1_ + 0.21·*X*_1_*X*_2_ + 0.46·*X*_1_^2^, (9)

*Y*_7_ = 28.2 − 4.59·*X*_1_ − 1.39·*X*_2_ + 1.39·*X*_1_*X*_2_ + 1.69·*X*_2_^2^, (10)

where *Y*_5_ represents the average density of concrete, *Y*_6_ represents the flexural strength, and *Y*_7_ represents the compressive strength. However, our analysis of the obtained mathematical models was complicated by the presence of segregated compositions at *X*_1_, and *X*_2_ tends to 1 of the experimental plan (Table 9). The equation *Y*_5_ = *f*(*X*_1_, *X*_2_) shows the statistically insignificant influence of variable factors on the average densities of the concretes. Only the amount of water is a significant factor, according to the model. In this case, the value of coefficient B_1_ shows the negative impact of the W/C ratio on the average densities of the concretes. This is explained by the delamination of concrete with an increase in the amount of water (W/C ≥ 0.6). An analysis of equations *Y*_6_ and *Y*_7_ allows one to conclude that water content has a similar effect on flexural and compressive strength (negative value of coefficient B_1_). The role of the plasticizer becomes more significant. This is reflected by a positive influence in quantities close to the boundaries of the variable range and the joint influence of *C*_Pl_ and W/C ratio (coefficients B_22_ and B_12_). The established experimental and statistical models highlight the need to optimize the studied compositions in terms of the W/C ratio and plasticizer concentration to obtain concrete with a homogeneous structure and high strength properties. Changes in physical and mechanical properties in the selected range of varying factors are in the following ranges: ρ = 1403–1485 kg/m^3^, *R*_fl_ = 3.34–5.90 MPa, *R*_com_ = 29.6–45.7 MPa.

Table 12 shows that the total content of the fractionated sand is not enough to significantly influence the effect of the dispersion (variation of the proportion of fine and fractionated sand S_p_/S_f_) of this component on the physical and mechanical properties of lightweight concrete. However, the highest values in terms of flexural and compressive strength were observed in the composition with S_p_/S_f_ = 50/50.

At the same time, since the complete absence of a thin or coarse part of the sand does not provide an optimal combination of these properties, optimizing the S_p_/S_f_ ratio is important. The flexural strength of the self-compacting lightweight concrete on hollow microspheres is 44–53% of the strength values of Prototype 1 and Prototype 2 and 45–66% of their compressive strength values. Moreover, the average density is 1.5 times less than that of the heavy concretes.

Thus, the ability of a concrete mixture based on hollow microspheres (average density 1450 ± 25 kg/m^3^) to self-compact has been established. The close values of the spread diameter before and after shaking on the table (*D*_sp,1_ tended to *D*_sp,2_) is confirmation of this. The intensity of the influence of the W/C ratio and plasticizer concentration on free flow diameter (*D*_sp,1_) is higher than that of the spreading diameter after shaking (*D*_sp,2_). The self-compacting ability of the lightweight concrete with hollow microspheres is higher than that of heavy concrete (Prototype 1) at the same W/C ratio and *C*_Pl_ (W/C = 0.5 and *C*_Pl_ = 1.4%).

An assessment of the flow rheology of the concrete mixtures using the coefficients of the Ostwald–Weil equation showed that the W/C ratio is a more significant factor in terms of its influence on the density (thickness) of the concrete mixture (*k*) than the concentration of the plasticizer. In this case, a decrease in the thickness of the dispersed systems under study can naturally be observed with an increase in both W/C ratio and *C*_Pl_. The obtained regression equations for the index n in the Ostwald–Weil equation show the possibility of changing the nature of the flow of mixtures from pseudoplastic (*n* < 1) to dilatant (*n* > 1) under varying W/C ratios and *C*_Pl_ values.

Mixtures of lightweight concrete with a content of hollow microspheres of 46.4% by volume do not have delamination at W/C ≤ 0.6 and *C*_Pl_ ≤ 1.0%. An increase in the W/C ratio or additive concentration leads to a violation of the homogeneity of the concrete mixture. The presence of an external influence on the mixture before molding shifts the limits of homogeneity for variable factors towards lower values. Changes in the physical and mechanical properties of lightweight concrete on hollow microspheres in the selected range of varying the W/C ratio and plasticizer concentration are in the following ranges: ρ = 1403–1485 kg/m^3^, *R*_fl_ = 3.34–5.90 MPa, *R*_com_ = 29.6–45.7 MPa. At the same time, the presence of delamination at W/C ≥ 0.6 does not allow one to correctly establish the influence of variable factors.

The dispersion of quartz sand (varying the S_p_/S_f_ ratio) in an amount of 8.7% in the composition of lightweight concrete does not have a significant effect on the self-compaction criterion *D*_sp,2_/*D*_sp,1_. Replacing fractionated sand with fine sand from 100 to 0% contributes to increasing the density by almost three-fold, which is naturally associated with a larger total surface area of the quartz part in the mixture. Concrete mixtures with different ratios of fine and fractionated sand (S_p_/S_f_) (at W/C = 0.5 and *C*_Pl_ = 1.4%) have similar homogeneity and tend to separate after a certain threshold of external influence. It has been shown that varying the ratio of fine and fractionated sand while maintaining their total volumetric contents (8.7%) in the composition of lightweight concrete does not have a significant effect on the physical and mechanical properties. The highest value of flexural and compression strength was observed in compositions with S_p_/S_f_ = 50/50.

In future research, to ensure the development of the topic of this study, researchers should aim to establish the influence of the studied factors on the strength properties in systems without delamination and search for and establish the parameters and boundary values of delamination after external influence on the concrete mixture before molding.

## 4. Conclusions

Carrying out this study allowed us to conclude that it is possible to produce self-compacting lightweight concrete using hollow microspheres. The key contributions and conclusions of this paper are as follows:

- The ability of a lightweight concrete mixture with a low density (ρ = 1450 ± 25 kg/m^3^) to self-compact has been established.
- The dependences (regression equations) of mobility, coefficients of the Ostwald–Weil equation, and density and strength od the W/C ratio and plasticizer concentration for lightweight concrete with a volume content of hollow microspheres of 46.4% have been established.
- The limits for homogeneity of lightweight concrete mixtures on hollow microspheres are W/C ≤ 0.6 and C_Pl_ ≤ 1.0%.
- The dispersion of quartz sand (varying the S_p_/S_f_ ratio) in an amount of 8.7% in the composition of lightweight concrete does not have a significant effect on the self-compaction criterion and physical and mechanical properties.
- This paper shows the possibility of changing the nature of the flow of lightweight concrete mixtures from pseudoplastic to dilatant under varying W/C ratios and plasticizer concentrations.

Future research directions for the development of this research topic include the following:

- Establishing the influence of the studied factors on the strength properties in systems without delamination.
- Establishing the parameters and boundary values of delamination after external influence on the concrete mixture.
- Studying the time it takes to maintain the mobility and homogeneity of the concrete mixture on hollow microspheres.

## Figures and Tables

**Table 1:** Chemical and mineralogical composition of clinker.

Oxides							Minerals			
CaO	SiO_2_	Al_2_O_3_	Fe_2_O_3_	MgO	SO_3_	Na_2_O	C_3_S	C_2_S	C_3_A	C_4_AF
66.0	21.2	5.1	4.1	0.75	0.56	0.58	68.2	8.2	6.4	12.6

**Table 2:** The main properties of the Portland cement.

No	Property	Value
1	Compressive strength (age is 2 days), MPa	22.9
2	Compressive strength (age is 28 days), MPa	52.6
3	Initial/finish setting time, min	175/300
4	Specific surface area, m^2^/kg	408
5	Normal density of cement dough, %	24.3

**Table 3:** Properties of hollow aluminosilicate microspheres (ForeSphere).

No	Property	Value
1	Bulk density, kg/m^3^	320–370
2	True density, kg/m^3^	580–690
3	Average particle size, μm	100
4	The thickness of the walls of the microsphere, μm	2–10
5	Wall material density, kg/m^3^	2500
6	Compressive strength, MPa	15.0–28.0
7	Mohs scale of mineral hardness	5–6

**Table 4:** Experiment planning matrix.

No	Coded Value		Natural Value	
	*X*_1_	*X*_2_	W/C	C_Pl_
1	−1	−1	0.40	1.00
2	+1	−1	0.60	1.00
3	−1	+1	0.40	1.80
4	+1	+1	0.60	1.80
5	−1.414	0	0.36	1.40
6	+1.414	0	0.64	1.40
7	0	−1.414	0.50	0.83
8	0	+1.414	0.50	1.97
9	0	0	0.50	1.40

**Table 5:** Ratio of components of the concrete mixtures.

No	Composition	Volume Content, %				
		PC	SA	S_p_	S_f_	MS
1	Lightweight concrete	20.0	3.1	2.2	6.5	46.4
2	Prototype 1 (heavy concrete)	20.0	3.1	14.1	41.0	0.0
3	Prototype 2 (heavy concrete)	20.0	3.1	14.1	41.0	0.0

**Table 6:** The volume content of the components of the concrete mixtures with varying S_p_/S_f_ ratios.

No	Component	Volume Content, %				
		0/100	25/75	50/50	75/25	100/0
1	Fine sand (powder) (S_p_)	0.0	2.2	4.35	6.5	8.7
2	Fractional sand (S_f_)	8.7	6.5	4.35	2.2	0.0

**Table 7:** Mobility parameters of heavy concrete mixtures.

No	Property	Value	
		Prototype 1	Prototype 2
1	Spread diameter before shaking, *D*_sp,1_, mm	279 ± 2	229 ± 2
2	Spread diameter after shaking, *D*_sp,2_, mm	302 ± 7	262 ± 7
3	Self-compacting coefficient, *D*_sp,2_/*D*_sp,1_	1.08	1.14

**Table 8:** Mobility parameters of lightweight concrete mixtures with various S_p_/S_f_ ratios.

No	Property	S_p_/S_f_				
		0/100	25/75	50/50	75/25	100/0
1	Spread diameter before shaking, *D*_sp,1_, mm	305 ± 6	304 ± 4	301 ± 5	305 ± 6	302 ± 5
2	Spread diameter after shaking, *D*_sp,2_, mm	317 ± 2	314 ± 5	313 ± 3	315 ± 5	317 ± 3
3	Self-compacting coefficient, *D*_sp,2_/*D*_sp,1_	1.04	1.03	1.04	1.03	1.05

**Table 9:** Coefficients of the Ostwald–Weil equation for the rheological flow curves of lightweight concrete mixtures when varying the ratio of fractional and fine quartz sand.

No	Property	S_p_/S_f_					Prototype 1	Prototype 2
		0/100	25/75	50/50	75/25	100/0		
1	*k*	35.4	49.9	57.7	62.0	102.9	31.2	77.7
2	*n*	0.94	0.97	0.94	0.97	1.07	0.68	0.63

**Table 10:** Segregation in lightweight concrete samples.

No	Composition	Value								
		1	2	3	4	5	6	7	8	9
	W/C	0.4	0.6	0.4	0.6	0.36	0.64	0.5	0.5	0.5
	*C*_Pl_, %	1.0	1.0	1.8	1.8	1.4	1.4	0.83	1.97	1.4
1	Series 1	No	No	No	+	No	+	No	No	No
2	Series 2	No	No	No	+	No	+	No	No	No
3	Series 3	No	+	No	+	No	+	+	+	+

Note: + shows the presence of visual signs of delamination.

**Table 11:** Segregation in lightweight concrete samples with various S_p_/S_f_ ratios.

No	Composition	S_p_/S_f_				
		0/100	25/75	50/50	75/25	100/0
1	Series 1	No	No	No	No	No
2	Series 2	No	No	No	No	No
3	Series 3	+	+	+	+	+

Note: + shows the presence of visual signs of delamination.

**Table 12:** Physico-mechanical properties of lightweight concretes on hollow microspheres.

S_p_/S_f_	Average Density, kg/m^3^	Flexural Strength, MPa	Compressive Strength, MPa
0/100	1420 ± 10	3.20 ± 0.10	23.6 ± 1.2
25/75	1410 ± 25	3.40 ± 0.10	28.1 ± 0.9
50/50	1420 ± 5	3.80 ± 0.05	31.5 ± 1.5
75/25	1445 ± 35	3.25 ± 0.15	28.0 ± 1.3
100/0	1410 ± 15	3.25 ± 0.45	28.3 ± 1.2
Prototype 1	2165 ± 10	7.50 ± 0.15	55.0 ± 2.4
Prototype 2	2105 ± 10	3.35 ± 0.20	47.1 ± 2.4

## Data Availability

Data are available upon request due to restrictions (e.g., privacy or ethical). The data presented in this study are available from the corresponding author upon request. The data are not publicly available due to commercial goals.
